# Decoding the Myocardium: Tracer-Aware Deep Learning for Patient-Level Classification in Stress–Rest SPECT Myocardial Perfusion Imaging

**DOI:** 10.3390/diagnostics16121796

**Published:** 2026-06-10

**Authors:** Dimitrios Samaras, Dimitra Tsivaka, Maria Vakalopoulou, Panagiotis Papadimitroulas, George Angelidis, Thomas Kilindris, Varvara Valotassiou, Dimitrios Psimadas, Emmanouil Panagiotidis, Panagiotis Georgoulias, Ioannis Tsougos

**Affiliations:** 1 Medical Physics Laboratory, Faculty of Medicine, University of Thessaly, 41500 Larissa, Greece; dimitsamaras@uth.gr (D.S.); dtsivaka@uth.gr (D.T.); 2 Archimedes Research Unit, Athena Research Center, 15125 Athens, Greece; maria.vakalopoulou@centralesupelec.fr; 3 Medical Informatics and Biomedical Imaging Laboratory, Faculty of Medicine, University of Thessaly, 41500 Larissa, Greece; ppapadimitroulas@uth.gr (P.P.); tomkil@uth.gr (T.K.); 4 MICS Laboratory, CentraleSupélec, Université Paris-Saclay, 91190 Gif-sur-Yvette, France; 5 Nuclear Medicine Laboratory, University Hospital of Larissa, University of Thessaly, 41110 Larissa, Greece; angelidis@protonmail.ch (G.A.); valotasiou@uth.gr (V.V.); dpsimad@chem.uoa.gr (D.P.); manospanagiotidis@uth.gr (E.P.); pgeorgoul@med.uth.gr (P.G.)

**Keywords:** SPECT MPI, polar maps, coronary artery disease, deep learning

## Abstract

**Background/Objectives:** Single-photon emission computed tomography (SPECT) myocardial perfusion imaging (MPI) is widely used for non-invasive assessment of coronary artery disease under stress and rest conditions. Although deep learning has shown promise for automated SPECT MPI interpretation, most studies focus on single-tracer datasets and do not explicitly account for tracer-dependent variability. This study developed and evaluated a multi-task deep learning framework with tracer-specific prediction heads for patient-level SPECT MPI classification. **Methods:** A convolutional neural network with a shared feature encoder and tracer-specific heads was implemented using polar map representations from technetium-99m (Tc-99m) and thallium-201 (Tl-201) studies. Transfer learning from ImageNet was applied. Stress-only, rest-only, and dual-input configurations were evaluated using repeated patient-stratified cross-validation and independent testing. Performance was assessed using ROC-AUC and balanced accuracy. **Results:** For Tc-99m normal versus abnormal perfusion classification, the stress-only model achieved the highest cross-validation AUC (0.88 ± 0.067) and test AUC of 0.88 [0.67–0.99]. For Tl-201 low-risk versus intermediate/high-risk classification, stress-based models achieved the highest cross-validation AUC (0.88 ± 0.051) and test AUC of 0.80 [0.71–0.89], comparable to dual-input models. In both tracer-specific tasks, stress-phase information showed favorable performance, but the endpoints differed and should be interpreted separately. **Conclusions:** Stress-phase polar maps provided strong discriminative information within this single-center cohort. These findings should be interpreted in a tracer- and task-specific manner supporting stress-phase imaging as an informative input for AI-based SPECT MPI classification while underscoring the need for external validation before broader clinical generalization.

## 1. Introduction

Single-photon emission computed tomography (SPECT) myocardial perfusion imaging (MPI) is a widely used non-invasive modality for the diagnosis, risk stratification, and longitudinal monitoring of patients with suspected or established coronary artery disease (CAD). SPECT MPI provides physiological information on regional myocardial blood flow by imaging the distribution of radiotracers preferentially taken up by viable myocardium [1]. In routine clinical practice, the most commonly used tracers include thallium-201 (Tl-201), which exhibits intracellular redistribution, and technetium-99m (Tc-99m)-labeled compounds (e.g., sestamibi, tetrofosmin), which demonstrate uptake patterns reflecting regional perfusion and cellular viability [2]. By capturing perfusion under both stress and rest conditions, SPECT MPI provides diagnostic and prognostic information that is well integrated into contemporary clinical workflows.

Early clinical investigations established the value of SPECT MPI for differentiating ischemic from non-ischemic myocardium and for characterizing myocardial injury and recovery following coronary interventions. A clinical study [3] involving patients with persistent symptoms after percutaneous coronary intervention demonstrated the utility of myocardial perfusion imaging in identifying inducible ischemia and reperfusion-related myocardial injury. Complementary methodological work has further consolidated the clinical role of SPECT MPI by outlining radiotracer protocols, acquisition techniques, and quantitative assessment principles, emphasizing the complementary use of Tl-201 and Tc-99m–based protocols in routine clinical practice [4].

In parallel, machine learning and deep learning methods have increasingly been applied to SPECT MPI with the aim of automating or augmenting interpretation, reducing inter-reader variability, and improving diagnostic consistency [5]. Convolutional neural networks (CNNs) have demonstrated promising performance in classifying perfusion abnormalities from stress and rest representations, including polar maps; however, most existing studies have focused on single-tracer datasets or narrowly defined imaging protocols [6]. Consequently, the impact of tracer heterogeneity and input configuration on model generalization remains insufficiently explored.

From a clinical perspective, an important and unresolved question is whether all available imaging information is necessary for accurate automated classification. In particular, although stress imaging is generally considered the most diagnostically informative phase, the extent to which rest data contributes to patient-level classification remains unclear. Furthermore, differences between tracers such as Tc-99m and Tl-201, which influence image characteristics, acquisition protocols, and clinical interpretation, are rarely explicitly incorporated into deep learning frameworks.

In this study, we propose a tracer-specific multi-task deep learning framework for patient-level SPECT MPI classification using polar map representations. A shared convolutional encoder with tracer-specific prediction heads is employed to capture both common and tracer-dependent imaging characteristics. Stress-only, rest-only, and dual-input configurations are evaluated under identical patient-level protocols in order to systematically assess the effect of input configuration and tracer variability on model performance.

The primary contribution of this study is a patient-level evaluation of stress-only, rest-only, and dual-input deep learning models for SPECT MPI using repeated patient-stratified cross-validation and independent testing. Specifically, this work systematically compares stress-only, rest-only, and dual-input stress–rest polar-map configurations under the same experimental protocol and evaluates a shared ResNet-18 feature encoder with tracer-specific prediction heads for Tc-99m and Tl-201 studies. By analyzing two tracer-specific clinical tasks separately, this study provides evidence on the relative discriminative contribution of stress-phase and rest-phase polar map information while accounting for tracer-associated differences within a common feature-learning framework.

## 2. Materials and Methods

### 2.1. Study Population and Data Collection

This retrospective study included 640 patients who underwent clinically indicated myocardial perfusion imaging (MPI) using single-photon emission computed tomography (SPECT). Imaging was performed under standard clinical protocols, with acquisitions obtained during both stress and rest conditions.

The study cohort comprised 439 male (68.6%) and 201 female (31.4%) patients. The mean age was comparable between sexes (64.9 years for males and 64.6 years for females). Male patients presented a higher mean body weight (86.6 kg) compared to female patients (76.8 kg).

Examinations were performed using technetium-99m (Tc-99m) and thallium-201 (Tl-201), with a total of 274 Tc-99m and 366 Tl-201 studies.

Following reconstruction, volumetric SPECT MPI data were processed according to the institutional clinical post-processing protocol and transformed into two-dimensional polar maps representing relative myocardial perfusion across the left ventricular myocardium under stress and rest conditions. Polar maps were selected due to their routine clinical use, compact representation, and ability to preserve diagnostically relevant perfusion patterns while enabling efficient computational analysis.

**Figure 1:**
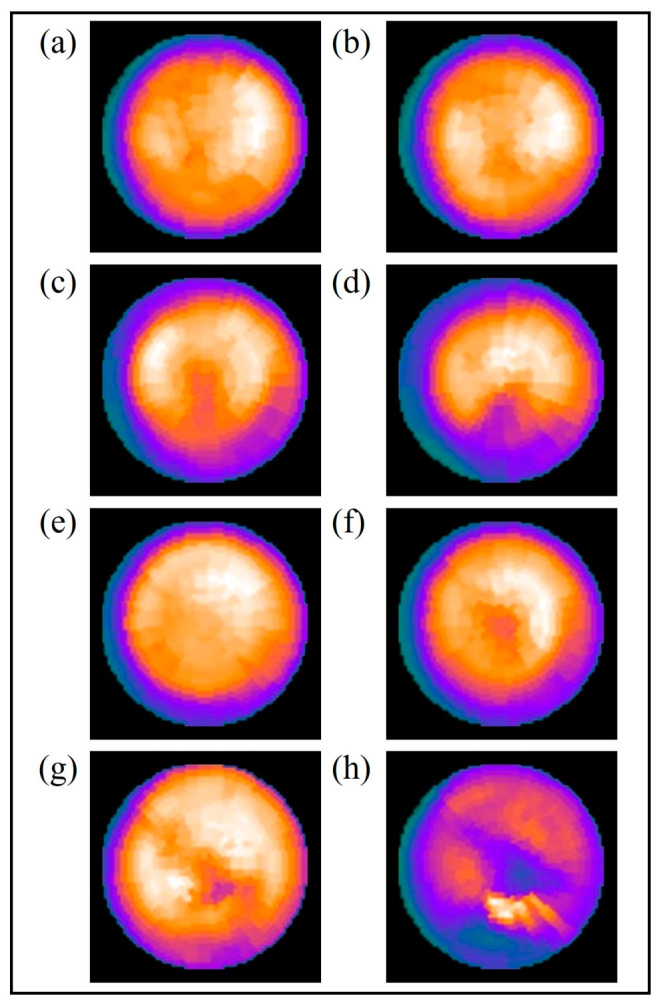
Representative rest and stress SPECT MPI polar maps from the two tracer-specific classification tasks. Panels (**a**,**b**) show a representative normal Tc-99m case at rest and stress, respectively; panels (**c**,**d**) show a representative abnormal Tc-99m case at rest and stress, respectively; panels (**e**,**f**) show a representative low-risk Tl-201 case at rest and stress, respectively; and panels (**g**,**h**) show a representative intermediate/high-risk Tl-201 case at rest and stress, respectively. These examples illustrate the rendered RGB polar-map format used as model input and the visual appearance of the task-specific reference categories. They are provided for qualitative visualization of the input data and do not constitute formal model interpretability analysis.

Representative stress-phase polar maps from the two tracer-specific tasks are shown in Figure 1. The examples illustrate the rendered RGB polar-map format used as model input and show corresponding reference labels and model-predicted probabilities for representative normal/abnormal Tc-99m and low-risk/intermediate-high-risk Tl-201 cases.

Patient demographics and tracer distribution are summarized in Table 1. The final dataset consisted of 274 Tc-99m examinations (229 normal and 45 abnormal) and 366 Tl-201 examinations (91 low-risk and 275 intermediate/high-risk). All analyses were conducted at the patient level to ensure clinical relevance and to prevent information leakage between training and evaluation phases.

The 80/20 patient-level split resulted in 512 patients in the training set and 128 patients in the independent test set. At the tracer-specific level, the independent test set included 55 Tc-99m patients, of whom 46 were normal and 9 were abnormal, and 73 Tl-201 patients, of whom 18 were low-risk and 55 were intermediate/high-risk. When multiple image representations or examinations were available for the same patient, all corresponding data were assigned to the same partition to prevent patient-level information leakage.

### 2.2. Ground Truth Definition

Ground truth labels for both tracer-specific tasks were established retrospectively from expert clinical evaluation and were assigned independently of the developed deep learning models. The expert assessment was based on the complete SPECT MPI examination together with the available clinical context recorded during routine patient evaluation.

For Tc-99m studies, the binary endpoint reflected the presence or absence of myocardial perfusion abnormality. The negative class represented normal perfusion, whereas the positive class represented abnormal perfusion findings. For Tl-201 studies, the binary endpoint reflected clinical risk stratification. Normal- and Low-risk examinations were assigned to the negative class, whereas intermediate- and high-risk examinations were grouped into the positive class. Reference labels were derived from expert interpretation of the complete SPECT MPI examination.

### 2.3. Model Development

**Figure 2:**
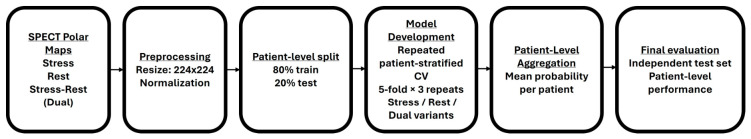
Patient-level experimental pipeline for SPECT MPI analysis.

An overview of the patient-level experimental pipeline is presented in Figure 2.

Polar maps were loaded as rendered RGB images, converted to three-channel tensors, and resized to 224 × 224 pixels. Channel-wise normalization was performed using ImageNet mean and standard deviation values to ensure compatibility with the ImageNet-pretrained ResNet-18 backbone. This normalization was selected because transfer learning was performed from an ImageNet-pretrained network and the polar maps were used as rendered RGB images; however, no separate comparison with domain-specific normalization strategies was performed. All polar maps were generated using the same institutional processing workflow, thereby maintaining consistent rendering and color-scale conventions within the present dataset.

Three input configurations were evaluated: stress-only, rest-only, and dual-input stress–rest. In the stress-only configuration, only the stress polar map was passed through the ResNet-18 encoder, producing a 512-dimensional feature vector [7]. In the rest-only configuration, only the rest polar map was passed through the same encoder architecture. In both single-input configurations, the resulting 512-dimensional feature vector was passed to the tracer-specific classification head.

In the dual-input configuration, stress and rest polar maps were not stacked as image channels. Instead, both images were processed separately by the same ResNet-18 encoder with shared weights, producing a 512-dimensional stress feature vector and a 512-dimensional rest feature vector. These vectors were fused by concatenating the stress representation, the rest representation, and their absolute feature-wise difference (Equation (1)), resulting in a 1536-dimensional representation:

z_dual_ = [z_stress_, z_rest_, |z_stress_ − z_rest_|], (1)

This fused representation was passed to the corresponding tracer-specific classification head.

Each tracer-specific classification head consisted of a fully connected layer with 256 units, ReLU activation, dropout of 0.2, and a final single-neuron output layer producing the binary logit. The ResNet-18 backbone was initialized with ImageNet-pretrained weights. During fine-tuning, all backbone layers were frozen except the final residual block, which was updated with a learning rate of 1 × 10^−5^. The tracer-specific classification heads were trained with a learning rate of 1 × 10^−4^. Optimization was performed using AdamW with weight decay of1 × 10^−4^, a batch size of 32, and 20 training epochs. No data augmentation was applied in the final experiments.

In this context, the term “tracer-aware” refers to the use of tracer-specific prediction heads within a shared multi-task architecture, rather than to the explicit inclusion of tracer identity as an additional numerical or categorical input feature.

Model training was performed using weighted binary cross-entropy loss to account for class imbalance between clinical categories. For the multi-task tracer-specific architecture, class weights were calculated separately for each tracer-specific task using only the corresponding training data within each training fold. Separate weighted loss terms were then applied to the Tc-99m and Tl-201 prediction heads. This strategy avoided the use of validation or test-set label distributions during optimization. Model selection was based on the highest mean validation ROC-AUC averaged across the two tracer-specific validation tasks. Following cross-validation, predictions were aggregated at the patient level by averaging probabilities across examinations from the same patient.

To prevent information leakage and ensure clinical validity, all data partitioning was performed at the patient level. The dataset was divided into a training set (80%, *n* = 512 patients) and an independent test set (20%, *n* = 128 patients) using stratified sampling based on tracer type and clinical label. Model development and selection were conducted exclusively on the training set using repeated patient-stratified cross-validation with five folds and three repeats. Patient identifiers were used as the grouping variable for both the hold-out split and cross-validation fold assignment. In all splitting procedures, all examinations and image representations belonging to the same patient were assigned to the same partition or fold, and no patient appeared in both the training and independent test sets.

To evaluate the contribution of tracer-specific prediction heads, an additional tracer-agnostic single-head CNN baseline was implemented as an ablation analysis. This baseline used the same ResNet-18 encoder, polar-map preprocessing pipeline, patient-level train/test split, repeated patient-stratified cross-validation protocol, fine-tuning strategy, optimizer, and training hyperparameters as the proposed multi-task model. However, instead of using separate Tc-99m and Tl-201 prediction heads, the baseline used a single shared output head for both tracers. Because only one output head was used, model training was performed with a global class-weighted binary cross-entropy loss. Predictions from the single-head baseline were evaluated separately for the Tc-99m and Tl-201 tasks.

**Figure 3:**
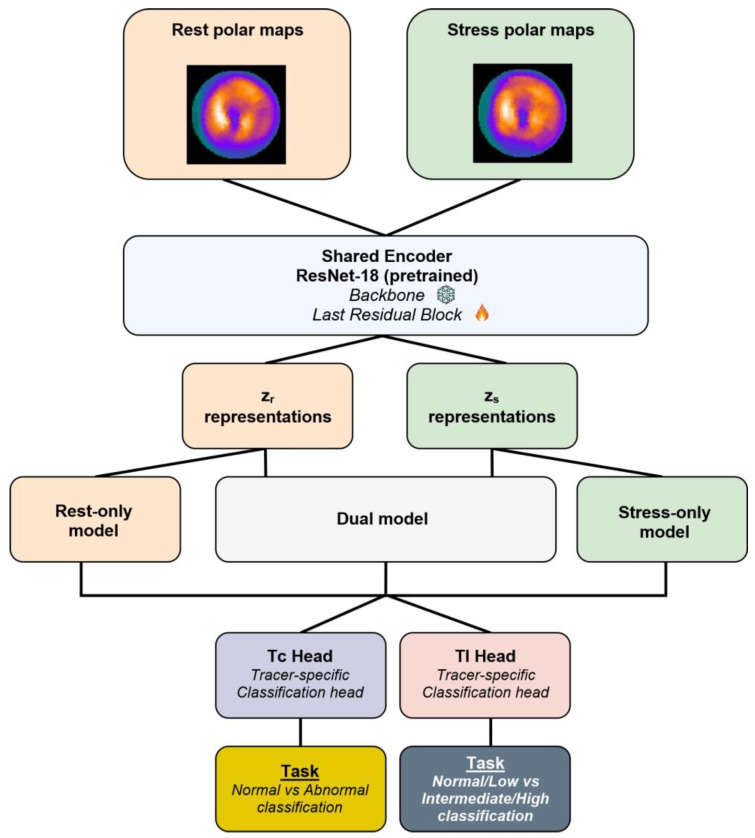
Multi-task deep learning architecture with tracer-specific prediction heads for patient-level SPECT MPI classification.

A schematic representation of the model architecture is shown in Figure 3.

### 2.4. Statistical Analysis

Model performance was evaluated at the patient level using ROC-AUC and balanced accuracy as primary metrics. Sensitivity and specificity were also computed as clinically relevant threshold-dependent metrics to support interpretation of model behavior. For independent test-set performance, 95% confidence intervals were estimated using 2000 patient-level bootstrap resampling iterations.

Pairwise comparisons between input configurations were performed using bootstrap-based differences in patient-level ROC-AUC. Comparisons were performed separately for each tracer-specific task and for both cross-validation and independent test-set predictions. For each comparison, patient-level predictions were resampled with replacement over 2000 bootstrap iterations. The ROC-AUC difference between each pair of input configurations was calculated in each bootstrap sample, and 95% confidence intervals were derived from the empirical bootstrap distribution. Two-sided *p*-values were estimated from the bootstrap distribution. The evaluated pairwise comparisons were stress-only versus dual-input, stress-only versus rest-only, and dual-input versus rest-only. These analyses were considered exploratory and were used to assess whether observed differences between input configurations were statistically supported. No formal non-inferiority margin was prespecified; therefore, the study does not claim statistical non-inferiority of stress-only models relative to dual-input models.

## 3. Results

### 3.1. Polar Map-Based Classification Performance

The classification performance of the evaluated models is summarized in Table 2 and Table 3, for both Tc-99m and Tl-201 tasks and across all input configurations (rest-only, stress-only, and dual-input). Performance is reported at the patient level. Cross-validation results are presented as mean ± standard deviation across 15 validation folds from five-fold cross-validation repeated three times, while independent test-set performance is reported with 95% confidence intervals.

### 3.2. Tc-99m: Normal Versus Abnormal Perfusion Classification

For Tc-99m studies, the stress-only model demonstrated the most consistent performance across both cross-validation and independent testing. During cross-validation, it achieved a mean AUC of 0.88 ± 0.067 and a balanced accuracy of 0.75 ± 0.061. On the independent test set, it yielded an AUC of 0.88 [0.67–0.99] and a balanced accuracy of 0.87 [0.70–0.98]. Independent test-set sensitivity and specificity for the stress-only Tc-99m model were 0.78 and 0.96, respectively. However, because the independent test set included a limited number of abnormal Tc-99m cases, these estimates should be interpreted cautiously.

**Table 2:** Patient-level classification performance for stress-only, rest-only, and dual-input models on Task 1 (Tc-99m: normal vs. abnormal perfusion classification task). Training results are reported as mean ± standard deviation across repeated cross-validation, while test results are reported with 95% confidence intervals.

Training			Test	
Model	AUC	Bal. Accuracy	AUC	Bal. Accuracy
Rest-only	0.75 ± 0.086	0.64 ± 0.071	0.85 [0.72, 0.94]	0.62 [0.46, 0.79]
Dual	0.86 ± 0.051	0.71 ± 0.084	0.91 [0.79, 0.99]	0.87 [0.70, 0.99]
Stress-only	0.88 ± 0.067	0.75 ± 0.061	0.88 [0.67, 0.99]	0.87 [0.70, 0.98]

The dual-input model showed a numerically higher test AUC of 0.91 [0.79–0.99] and a balanced accuracy of 0.87 [0.70–0.99], although formal pairwise comparisons did not show a statistically significant difference between dual-input and stress-only models on the independent test set. In contrast, the rest-only configuration demonstrated lower performance, with a cross-validation AUC of 0.75 ± 0.086 and test balanced accuracy of 0.62 [0.46–0.79].

**Figure 4:**
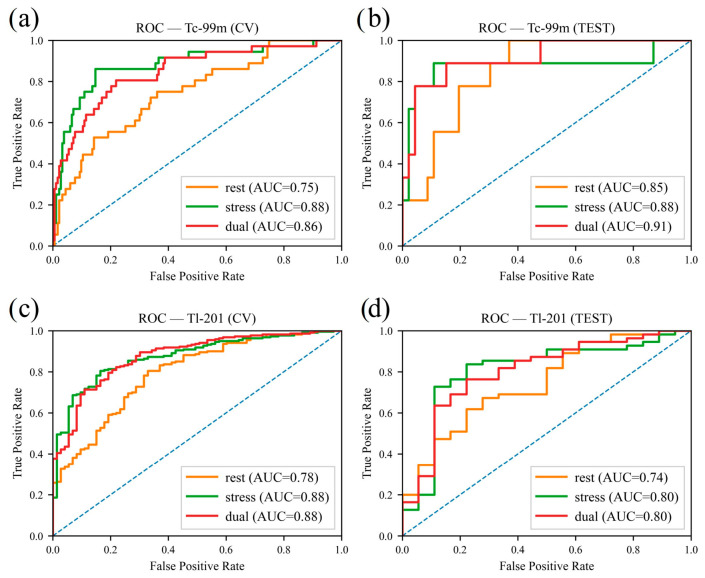
(**a**–**d**) Patient-level ROC curves for Tc-99m and Tl-201 tasks under cross-validation and independent testing, comparing rest-only, stress-only, and dual-input polar map CNN models.

Receiver operating characteristic (ROC) curves for Tc-99m are presented in Figure 4a (cross-validation) and Figure 4b (test set).

### 3.3. Tl-201: Low-Risk Versus Intermediate/High-Risk Classification

For Tl-201 studies, classification performance was lower overall. The stress-only model achieved a cross-validation AUC of 0.88 ± 0.051 and balanced accuracy of 0.78 ± 0.083, with corresponding test values of 0.80 [0.71–0.89] and 0.80 [0.68–0.89], respectively. Independent test-set sensitivity and specificity for the stress-only Tl-201 model were 0.82 and 0.78, respectively.

The dual-input model demonstrated similar performance, with a test AUC of 0.80 [0.67–0.91] and balanced accuracy of 0.76 [0.64–0.86]. The rest-only configuration showed reduced performance, particularly for test AUC (0.74 [0.61–0.87]) and balanced accuracy (0.66 [0.54–0.79]) on the test set.

ROC curves for Tl-201 are shown in Figure 4c (cross-validation) and Figure 4d (test set).

In both tracer-specific analyses, models incorporating stress-phase information showed favorable performance compared with rest-only models. However, because the Tc-99m and Tl-201 tasks used different clinical endpoints, these findings should be interpreted separately for each task rather than as a direct cross-tracer comparison.

**Table 3:** Patient-level classification performance for stress-only, rest-only, and dual-input models on Task 2 (Tl-201: low vs. intermediate/high risk classification task). Training results are reported as mean ± standard deviation across repeated cross-validation, while test results are reported with 95% confidence intervals.

Training			Test	
Model	AUC	Bal. Accuracy	AUC	Bal. Accuracy
Rest-only	0.78 ± 0.077	0.71 ± 0.082	0.74 [0.61, 0.87]	0.66 [0.54, 0.79]
Dual	0.88 ± 0.052	0.76 ± 0.087	0.80 [0.67, 0.91]	0.76 [0.64, 0.86]
Stress-only	0.88 ± 0.051	0.78 ± 0.083	0.80 [0.71, 0.89]	0.80 [0.68, 0.89]

### 3.4. Bootstrap-Based Pairwise Comparison of Input Configurations

Bootstrap-based pairwise comparisons of ROC-AUC differences between input configurations are presented in Table 4. Pairwise comparisons were based on 219 Tc-99m and 293 Tl-201 out-of-fold patient-level predictions during cross-validation, and 55 Tc-99m and 73 Tl-201 patient-level predictions in the independent test set.

In cross-validation, stress-only and dual-input configurations significantly outperformed rest-only models for both tracer-specific tasks. For Tc-99m, stress-only outperformed rest-only by ΔAUC = 0.134 (95% CI: 0.060 to 0.210, *p* < 0.001), while dual-input outperformed rest-only by ΔAUC = 0.102 (95% CI: 0.040 to 0.172, *p* = 0.004). For Tl-201, stress-only outperformed rest-only by ΔAUC = 0.080 (95% CI: 0.022 to 0.138, *p* = 0.006), while dual-input outperformed rest-only by ΔAUC = 0.082 (95% CI: 0.032 to 0.131, *p* < 0.001). No statistically significant difference was observed between stress-only and dual-input models in cross-validation for either Tc-99m or Tl-201.

On the independent test set, no statistically significant AUC difference was observed between input configurations for either tracer-specific task. For Tc-99m, the comparison between stress-only and dual-input models yielded ΔAUC = −0.036 (95% CI: −0.140 to 0.036, *p* = 0.579). For Tl-201, the corresponding comparison yielded ΔAUC = 0.004 (95% CI: −0.042 to 0.051, *p* = 0.895). These findings indicate that, on the independent test set, dual-input models did not demonstrate a statistically clear improvement over stress-only models in this cohort. However, because the test-set confidence intervals were wide, particularly for the Tc-99m task, these results should not be interpreted as evidence of equivalence or non-inferiority.

Values are reported as ΔAUC, 95% CI, and *p*-value. ΔAUC was calculated as the ROC-AUC of the first model minus the ROC-AUC of the second model. Confidence intervals and *p*-values were estimated using 2000 patient-level bootstrap resampling iterations. CV comparisons were based on out-of-fold patient-level predictions. No formal non-inferiority margin was prespecified.

### 3.5. Ablation Analysis of Tracer-Specific Prediction Heads

To assess the contribution of tracer-specific prediction heads, a stress-only ablation analysis was compared the proposed multi-task architecture with a tracer-agnostic single-head CNN baseline. The ablation was performed for the stress-only configuration because it showed the most favorable performance across the two tracer-specific tasks. The baseline used the same ResNet-18 encoder, preprocessing pipeline, patient-level split, repeated patient-stratified cross-validation protocol, and fine-tuning strategy, but mapped both Tc-99m and Tl-201 studies to a single shared output head. Predictions were evaluated separately for each tracer-specific task. The results of this ablation analysis are summarized in Table 5.

The single-head baseline achieved overall discrimination performance comparable to the tracer-specific-head model. For Tc-99m, the single-head model achieved a cross-validation AUC of 0.905 ± 0.066 and test AUC of 0.884 [0.680–0.997], compared with 0.885 ± 0.068 and 0.877 [0.670–0.998] for the tracer-specific-head model. For Tl-201, the single-head model achieved a cross-validation AUC of 0.873 ± 0.060 and test AUC of 0.792 [0.644–0.911], compared with 0.877 ± 0.051 and 0.805 [0.667–0.922] for the tracer-specific-head model.

Although AUC differences were small, the tracer-specific-head model showed more favorable balanced accuracy for Tl-201 during cross-validation (0.777 ± 0.083 versus 0.712 ± 0.107) and higher test sensitivity for Tl-201 (0.818 versus 0.691). These findings suggest that, while a single shared output head can achieve comparable overall discrimination, tracer-specific prediction heads provide a clinically motivated multi-task model that preserves separate decision pathways for Tc-99m and Tl-201 without compromising performance.

The single shared-head baseline used the same ResNet-18 encoder and stress-only training protocol but one output head for both tracers. Test-set confidence intervals were estimated using 2000 patient-level bootstrap resampling iterations. Balanced accuracy was calculated using thresholds calibrated from out-of-fold training predictions.

## 4. Discussion

This study investigated patient-level classification in myocardial perfusion imaging using SPECT polar maps, comparing stress-only, rest-only, and dual-input deep learning configurations across two clinically distinct tracers (Tc-99m and Tl-201). In both evaluated tasks, models incorporating stress-phase information achieved favorable performance compared with rest-only configurations. However, the Tc-99m and Tl-201 analyses corresponded to different clinical endpoints: normal versus abnormal perfusion for Tc-99m and low-risk versus intermediate/high-risk classification for Tl-201. Therefore, the findings should be interpreted in a task-specific manner and should not be considered evidence of endpoint-independent superiority of stress-phase imaging.

The heterogeneity of clinical endpoints is an important consideration in interpreting these results. The Tc-99m task reflects binary detection of perfusion abnormality, whereas the Tl-201 task reflects clinical risk stratification. These endpoints differ not only in clinical interpretation but also in the degree of uncertainty associated with the reference labels. As a result, direct numerical comparisons between the two tracer-specific tasks should be avoided. The present findings indicate that stress-phase polar maps were informative within each evaluated task, but they do not establish that stress-only imaging is equally sufficient across all SPECT MPI clinical endpoints.

This work provides a systematic evaluation of myocardial perfusion classification across two widely used SPECT tracers within a unified modeling framework. Prior deep learning studies in cardiac SPECT have predominantly focused on single-tracer Tc-99m imaging [8,9]. By introducing tracer-specific prediction heads on top of a shared feature encoder and leveraging transfer learning from large-scale natural image datasets, the proposed approach enables effective learning despite moderate dataset size and class imbalance. Conceptually, this design follows the principle of multi-task learning, in which related tasks share a common representation while task-specific output layers preserve differences in label definition, imaging characteristics, or clinical interpretation. The performance observed on the independent test set supports the feasibility of this strategy within the present cohort, although external validation is required to assess performance across different institutions and different acquisition protocols [10,11].

Recent deep learning studies in SPECT MPI have demonstrated the feasibility of automated CAD diagnosis and risk classification using polar maps, stress-only images, or reconstructed perfusion representations. Papandrianos et al. and Apostolopoulos et al. investigated CNN-based approaches for automated characterization of myocardial perfusion polar maps [8,9], while Liu et al. demonstrated that deep learning could improve diagnostic performance using stress-only SPECT MPI [10]. More recently, Wang et al. directly compared stress-only and rest–stress SPECT MPI using machine-learning approaches [11], and Kusumoto et al. developed an automated deep learning system for SPECT MPI diagnosis [12]. In contrast to these prior studies, the present work focuses specifically on patient-level comparison of stress-only, rest-only, and dual-input polar-map configurations across two tracer-specific clinical tasks. Furthermore, by using a shared encoder with tracer-specific prediction heads, the proposed framework explicitly accounts for tracer-associated differences within a unified validation protocol. Therefore, the contribution of this study lies not only in automated classification, but also in the systematic evaluation of input configuration and tracer-specific modeling in SPECT MPI.

The ablation analysis further clarified the role of tracer-specific prediction heads. The tracer-agnostic single-head CNN baseline achieved comparable AUC performance to the tracer-specific-head architecture, indicating that the shared encoder can learn relevant perfusion representations across tracers. However, the tracer-specific-head model showed more favorable balanced accuracy for Tl-201 during cross-validation and higher Tl-201 test sensitivity, while maintaining comparable overall discrimination. Given the differences in radiopharmaceutical pharmacokinetics, redistribution behavior, image characteristics, and clinical endpoint definition, the tracer-specific-head architecture remains methodologically justified as a clinically motivated multi-task formulation that preserves separate decision pathways without reducing performance.

Therefore, the tracer-aware nature of the proposed framework should be understood as architectural tracer awareness, implemented through separate Tc-99m and Tl-201 prediction heads, rather than as direct conditioning of the model on tracer labels.

In both tracer-specific tasks, stress-only and dual-input models showed favorable performance compared with rest-only configurations, particularly in cross-validation. Bootstrap-based pairwise comparisons showed that both stress-only and dual-input models significantly outperformed rest-only models in cross-validation, whereas stress-only and dual-input models did not differ significantly from each other. On the independent test set, no statistically significant AUC differences were observed between input configurations; therefore, these findings should be interpreted cautiously and should not be considered evidence of equivalence or non-inferiority [13]. The standard deviations observed across repeated cross-validation folds indicate fold-to-fold variability, particularly in the smaller Tc-99m abnormal subgroup, and should be considered when interpreting apparent numerical differences between input configurations.

From a clinical perspective, these results align with the established role of stress imaging as the primary component in the evaluation of myocardial perfusion. Stress-phase SPECT MPI reflects inducible ischemia and hemodynamic abnormalities, which are directly associated with clinically significant coronary artery disease [14]. The limited incremental benefit observed from the inclusion of rest information suggests that, within the evaluated framework and dataset, rest-phase data did not provide a clear additional discriminatory gain beyond stress-phase polar maps [12].

Importantly, the comparable performance between stress-only and dual-input models should be interpreted strictly within the context of the evaluated AI-based classification framework. These findings do not imply equivalence between stress-only and conventional rest–stress clinical protocols, and they should not be interpreted as suggesting that rest imaging is unnecessary in clinical practice. Rest imaging remains clinically important for comprehensive interpretation, including differentiation of attenuation artifacts from true perfusion abnormalities, assessment of fixed versus reversible defects, and evaluation of complex patterns such as multivessel disease [4,14,15,16]. Therefore, the present findings should be viewed as evidence that stress-phase polar maps were highly informative for the evaluated classification tasks, rather than as support for replacing rest imaging in routine SPECT MPI protocols.

From a practical standpoint, the favorable performance of stress-only models suggests that stress-phase polar maps may be useful when developing simplified AI-based triage or decision-support tools [17]. However, these potential workflow implications should be interpreted cautiously. The present study was not designed to evaluate clinical stress-only imaging protocols or to determine whether rest acquisitions can be omitted. Any clinical implementation would require prospective validation and external testing.

Methodologically, these findings suggest that increasing input dimensionality did not clearly improve model performance in the present cohort. Despite incorporating additional rest information, dual-input models did not consistently outperform simpler stress-only configurations. This underscores the importance of aligning model complexity with dataset characteristics and avoiding unnecessary multimodal integration when the incremental value is limited [13]. In this context, simpler architectures focusing on the most informative imaging phase may offer practical advantages in terms of simplicity and reproducibility.

Several limitations should be acknowledged. First, the dataset size constrained the ability to explore more granular clinical stratification through multiclass classification, particularly within the smaller Tc-99m abnormal subgroup. The limited number of positive Tc-99m cases in the independent test set also reduced the statistical power of pairwise model comparisons and contributed to wide confidence intervals.

Second, the study was conducted on a retrospective single-center dataset, and external validation across multiple institutions, imaging systems and acquisition protocols is required to assess generalizability. The use of rendered RGB polar maps is another limitation. Although this representation reflects the visual format used in routine clinical review and was generated consistently within the present single-center dataset, RGB color values do not directly represent raw quantitative tracer uptake. In addition, polar-map color scales and display conventions may vary across software packages, vendors, and institutions. Therefore, the robustness and transferability of models trained on RGB polar maps may be affected by differences in polar-map rendering. Future work should evaluate standardized quantitative polar maps or normalized perfusion-value maps and externally validate the approach across different systems and processing pipelines.

Third, the two tracer-specific analyses were based on different clinical endpoints. Tc-99m studies were labeled according to normal versus abnormal perfusion, whereas Tl-201 studies were labeled according to low-risk versus intermediate/high-risk classification. This endpoint heterogeneity limits direct cross-tracer comparison and requires cautious interpretation of any general conclusions regarding stress-only or dual-input model performance.

Another important limitation concerns the reference standard. The labels for both tracer-specific tasks were derived from expert assessment of the complete SPECT MPI examination together with the available clinical context. Therefore, the reference labels may partly incorporate information from the same imaging findings used as model inputs, including stress-phase information. This creates a potential source of incorporation bias when interpreting the favorable performance of stress-only models, particularly for the Tl-201 risk-stratification task. A sensitivity analysis using a fully independent reference standard, such as invasive coronary angiography, coronary CT angiography, or longitudinal clinical outcomes, was not feasible because such data were not systematically available for the full retrospective cohort. Future studies should validate these findings against independent anatomical or prognostic endpoints.

A further limitation is that a targeted error analysis of clinically important subgroups, such as attenuation artifacts and multivessel disease, was not feasible because these annotations were not systematically available in the retrospective dataset. This limits our ability to determine whether stress-only models are more likely to fail in scenarios where rest imaging provides essential confirmatory information. Future studies should include structured subgroup annotations to evaluate model behavior in clinically relevant cases where rest imaging remains diagnostically important.

Finally, the analysis was restricted to polar map representations. Future work should investigate the integration of tomographic perfusion images (short-axis, horizontal long-axis, vertical long-axis) and the incorporation of radiomics features and clinical variables to further enhance performance and clinical applicability. In addition, model interpretability was not evaluated in the present study. Future work should incorporate visualization techniques such as Grad-CAM, attention maps, or related explainability approaches to identify the imaging patterns driving model predictions, assess whether the model focuses on clinically meaningful myocardial regions, and support clinical trustworthiness.

## 5. Conclusions

This study evaluated stress-only, rest-only, and dual-input SPECT MPI polar-map models for two tracer-specific patient-level classification tasks using a multi-task deep learning architecture with tracer-specific prediction heads. The first main finding is that stress-phase polar maps provided the strongest and most consistent discriminative information within both tracer-specific tasks. The second main finding is that adding rest-phase information through a dual-input architecture did not provide a statistically clear improvement over stress-only input in this cohort, based on bootstrap-based ROC-AUC comparisons. The third contribution is the implementation and evaluation of a tracer-specific multi-task framework, which preserved separate decision pathways for Tc-99m and Tl-201 while maintaining performance comparable to a tracer-agnostic baseline. These findings should be interpreted in a tracer- and task-specific manner and should not be considered evidence that rest imaging is clinically unnecessary. Overall, the results support further investigation of stress-focused and tracer-aware AI approaches for SPECT MPI, while emphasizing the need for prospective and external validation before broader clinical generalization.

## Figures and Tables

**Table 1:** Baseline patient demographics and distribution of radiotracers in the study cohort.

Variable	Total (*n* = 640)
Sex (Male/Female)	439 (68.6%)/201 (31.4%)
Age (years, mean)	Male: 64.9/Female: 64.6
Weight (kg, mean)	Male: 86.6/Female: 76.8
Radiotracer (Tc-99m/Tl-201)	274/366

**Table 4:** Bootstrap-based pairwise ROC-AUC comparisons between input configurations.

Evaluation	Task	ΔAUC (Stress—Dual)	ΔAUC (Stress—Rest)	ΔAUC (Dual—Rest)
CV	Tc-99m	0.031 [−0.021, 0.084] *p* = 0.253	0.134 [0.060, 0.210] *p* < 0.001	0.102 [0.040, 0.172] *p* = 0.004
	Tl-201	−0.002 [−0.028, 0.025] *p* = 0.889	0.080 [0.022, 0.138] *p* = 0.006	0.082 [0.032, 0.131] *p* < 0.001
Test	Tc-99m	−0.036 [−0.140, 0.036] *p* = 0.579	0.029 [−0.192, 0.194] *p* = 0.714	0.065 [−0.048, 0.187] *p* = 0.263
	Tl-201	0.004 [−0.042, 0.051] *p* = 0.895	0.060 [−0.050, 0.172] *p* = 0.312	0.056 [−0.039, 0.151] *p* = 0.260

**Table 5:** Stress-only ablation comparing tracer-specific prediction heads with a tracer-agnostic single-head CNN baseline.

Architecture	Task	CV AUC	CV Bal. Accuracy	Test AUC	Test Bal. Accuracy
Tracer-specific heads	Tc-99m	0.88 ± 0.067	0.75 ± 0.061	0.88 [0.67, 0.99]	0.87 [0.70, 0.98]
Single shared head	Tc-99m	0.90 ± 0.066	0.80 ± 0.071	0.88 [0.68, 0.99]	0.84 [0.68, 0.98]
Tracer-specific heads	Tl-201	0.88 ± 0.051	0.78 ± 0.083	0.80 [0.71, 0.89]	0.80 [0.68, 0.89]
Single shared head	Tl-201	0.87 ± 0.060	0.71 ± 0.11	0.79 [0.64, 0.91]	0.79 [0.68, 0.87]

## Data Availability

The data presented in this study are available on request from the corresponding author due to privacy and ethical restrictions.

## Abbreviations

The following abbreviations are used in this manuscript:

AUC	Area Under the Curve
CAD	Coronary Artery Disease
CNNs	Convolutional Neural Networks
MPI	Myocardial Perfusion Imaging
ROC curves	Receiver Operating Characteristic curves
SPECT	Single Photon Emission Computed Tomography
Tc-99m	Technetium-99m
Tl-201	Thalium-201

## References

[1] Sun R., Ma R., Wang M., Han K., Zhang Z., Wang L., Fang W. (2023). Prognostic Value of Myocardial Flow Reserve Derived by Quantitative SPECT for Patients with Intermediate Coronary Stenoses. J. Nucl. Cardiol..

[2] Nappi C., Panico M., Falzarano M., Vallone C., Ponsiglione A., Cutillo P., Zampella E., Petretta M., Cuocolo A. (2023). Tracers for Cardiac Imaging: Targeting the Future of Viable Myocardium. Pharmaceutics.

[3] Georgoulias P., Valotassiou V., Tsougos I., Demakopoulos N. (2010). Myocardial Perfusion SPECT Imaging in Patients after Percutaneous Coronary Intervention. Curr. Cardiol. Rev..

[4] Georgoulias P., Angelidis G., Zisimopoulos A., Tsougos I., Georgoulias P.A., Angelidis G.C., Zisimopoulos A.S., Tsougos I.C. (2016). Myocardial Perfusion (SPECT) Imaging: Radiotracers and Techniques. Frontiers in Heart Failure.

[5] Angelidis G., Valotassiou V., Tsougos I., Tzavara C., Psimadas D., Theodorou E., Ziaka A., Giannakou S., Ziangas C., Skoularigis J. (2022). Automated Analysis vs. Expert Reading in Nuclear Cardiology: Correlations with the Angiographic Score. Medicina.

[6] Hajianfar G., Gharibi O., Sabouri M., Mohebi M., Amini M., Yasemi M.J., Chehreghani M., Maghsudi M., Mansouri Z., Edalat-Javid M. (2025). Artificial Intelligence-Powered Coronary Artery Disease Diagnosis from SPECT Myocardial Perfusion Imaging: A Comprehensive Deep Learning Study. Eur. J. Nucl. Med. Mol. Imaging.

[7] He K., Zhang X., Ren S., Sun J. Deep Residual Learning for Image Recognition. Proceedings of the IEEE Conference on Computer Vision and Pattern Recognition.

[8] Papandrianos N., Papageorgiou E. (2021). Automatic Diagnosis of Coronary Artery Disease in SPECT Myocardial Perfusion Imaging Employing Deep Learning. Appl. Sci..

[9] Apostolopoulos I.D., Papathanasiou N.D., Spyridonidis T., Apostolopoulos D.J. (2020). Automatic Characterization of Myocardial Perfusion Imaging Polar Maps Employing Deep Learning and Data Augmentation. Hell. J. Nucl. Med..

[10] Liu H., Wu J., Miller E.J., Liu C., Liu Y., Liu Y.-H. (2021). Diagnostic Accuracy of Stress-Only Myocardial Perfusion SPECT Improved by Deep Learning. Eur. J. Nucl. Med. Mol. Imaging.

[11] Wang F., Yuan H., Lv J., Han X., Zhou Z., Lu W., Lu L., Jiang L. (2024). Stress-Only versus Rest-Stress SPECT MPI in the Detection and Diagnosis of Myocardial Ischemia and Infarction by Machine Learning. Nucl. Med. Commun..

[12] Kusumoto D., Akiyama T., Hashimoto M., Iwabuchi Y., Katsuki T., Kimura M., Akiba Y., Sawada H., Inohara T., Yuasa S. (2024). A Deep Learning-Based Automated Diagnosis System for SPECT Myocardial Perfusion Imaging. Sci. Rep..

[13] Amini M., Pursamimi M., Hajianfar G., Salimi Y., Saberi A., Mehri-Kakavand G., Nazari M., Ghorbani M., Shalbaf A., Shiri I. (2023). Machine Learning-Based Diagnosis and Risk Classification of Coronary Artery Disease Using Myocardial Perfusion Imaging SPECT: A Radiomics Study. Sci. Rep..

[14] Gowd B.M.P., Heller G.V., Parker M.W. (2014). Stress-Only SPECT Myocardial Perfusion Imaging: A Review. J. Nucl. Cardiol..

[15] Otaki Y., Singh A., Kavanagh P., Miller R.J.H., Parekh T., Tamarappoo B.K., Sharir T., Einstein A.J., Fish M.B., Ruddy T.D. (2022). Clinical Deployment of Explainable Artificial Intelligence of SPECT for Diagnosis of Coronary Artery Disease. JACC Cardiovasc. Imaging.

[16] Fathala A. (2011). Myocardial Perfusion Scintigraphy: Techniques, Interpretation, Indications and Reporting. Ann. Saudi Med..

[17] Papandrianos N.I., Feleki A., Moustakidis S., Papageorgiou E.I., Apostolopoulos I.D., Apostolopoulos D.J. (2022). An Explainable Classification Method of SPECT Myocardial Perfusion Images in Nuclear Cardiology Using Deep Learning and Grad-CAM. Appl. Sci..

